# Bayesian multitask learning for medicine recommendation based on online patient reviews

**DOI:** 10.1093/bioinformatics/btad491

**Published:** 2023-08-08

**Authors:** Yichen Cheng, Yusen Xia, Xinlei Wang

**Affiliations:** Institute for Insight, Robinson College of Business, Georgia State University, Atlanta, GA 30303, United States; Institute for Insight, Robinson College of Business, Georgia State University, Atlanta, GA 30303, United States; Department of Mathematics, University of Texas at Arlington, Arlington, TX 76019, United States; Center for Data Science Research and Education, College of Science, University of Texas at Arlington, Arlington, TX 76019, United States

## Abstract

**Motivation:**

We propose a drug recommendation model that integrates information from both structured data (patient demographic information) and unstructured texts (patient reviews). It is based on multitask learning to predict review ratings of several satisfaction-related measures for a given medicine, where related tasks can learn from each other for prediction. The learned models can then be applied to new patients for drug recommendation. This is fundamentally different from most recommender systems in e-commerce, which do not work well for new customers (referred to as the cold-start problem). To extract information from review texts, we employ both topic modeling and sentiment analysis. We further incorporate variable selection into the model via Bayesian LASSO, which aims to filter out irrelevant features. To our best knowledge, this is the first Bayesian multitask learning method for ordinal responses. We are also the first to apply multitask learning to medicine recommendation. The sample code and data are made available at GitHub: https://github.com/thrushcyc-github/BMull.

**Results:**

We evaluate the proposed method on two sets of drug reviews involving 17 depression/high blood pressure-related drugs. Overall, our method performs better than existing benchmark methods in terms of accuracy and AUC (area under the receiver operating characteristic curve). It is effective even with a small sample size and only a few available features, and more robust to possible noninformative covariates. Due to our model explainability, insights generated from our model may work as a useful reference for doctors. In practice, however, a final decision should be carefully made by combining the information from the proposed recommender with doctors’ domain knowledge and past experience.

**Availability and implementation:**

The sample code and data are publicly available at GitHub: https://github.com/thrushcyc-github/BMull.

## 1 Introduction

In the USA, spending on prescription drugs is high and continues to increase. In 2020, the USA spent $4.1 trillion, or 19.7% of the gross domestic product, on national health expenditures, of which $348.4 billion was spent on prescription drugs (NHE Fact Sheet 2020). Over the next decade, the Centers for Medicare and Medicaid Services (CMS) projects that spending for retail prescription drugs will be the fastest growth health category and will consistently outpace that of other health spending (https://www.iqvia.com/insights/the-iqvia-institute/reports/medicine-spending-and-affordability-in-the-us). To treat a given disease, there may exist a handful of drug options, each with its pros and cons. Different drugs might be more suitable for different patient groups. To recommend the drug that is best suited for a person diagnosed with a certain disease is a challenging yet important task (Bartlett *et al.* 2000).

With the fast development of data collection and data analysis techniques for both structured and unstructured data, social media and online forums become great data resources for such tasks. In this article, we collect drug review data from an online forum, called WebMD, which is one of the most popular sites for patient reviews. For each reviewer (patient), we have star ratings on three measures (satisfaction level, effectiveness, and ease of use), age, gender, treatment time, and text review content. The collected information may enable us to obtain basic information about patients’ health status as well as key characteristics of each drug, all of which constitute an informative knowledge base toward better medicine recommendation.

Consistent with the recent research on data mining using social media data, we develop and evaluate a novel predictive framework in the context of medicine recommendation. In particular, we propose a Bayesian multitask learning (BMul) approach to predict patients’ ratings of three satisfaction-related measures for a given medicine. From an application perspective, the proposed approach makes several contributions. Firstly, unlike the existing approaches that might depend on very detailed medical and genetic information, which is usually not easy to obtain, our approach can provide personalized recommendation by making use of commonly available information upon a patient’s first visit. Such an information-light approach may augment the applicability of medicine recommendation well and greatly benefit both patients and doctors. Secondly, existing methods often focus on one measure of each medicine, and thus ignore the underlying connection among the different measures and some patients’ specific emphasis on some measure(s) over the other measures. The proposed approach combines the prediction of related measures into a unified framework and thus allows the models for different measures to “learn from each other” and thus achieve better performance. Thirdly, by extracting drug features from patient review contents using topic modeling and formally incorporating variable selection through Bayesian LASSO (Park and Casella 2008) to filter irrelevant topics, our approach can utilize text information efficiently and further make recommendation by considering a patient’s personal preference about treatment and side effects in addition to his health status. From a methodological perspective, we propose a BMul approach for ordinal regression with a variable selection component using a Laplace prior. Based on a latent variable view of the ordinal probit model, efficient sampling algorithms are implemented via a Gibbs sampler for both the original BMul model and the one with a shrinkage prior. To the best of our knowledge, this is the first BMul method designed for ordinal response variables.

We compare the proposed method (BMul) with several popular classification methods and show the advantage of BMul. In particular, we show that BMul has higher predictive power as compared to the competing methods, especially when the sample size is small. Thus, BMul can be used for new drugs with a limited number of reviews.

## 2 Research background

### 2.1 Medicine recommendation

Recent developments on medicine recommendation can be categorized into two classes according to their different setups. The first class assumes that the type of disease is known and aims to recommend a single drug that is deemed as the most appropriate treatment based on some prediction method, out of a list of drugs for treating a given health condition. This is similar to our problem setting. Palanivinayagam and Sasikumar (2020) proposed a drug recommendation system aiming at minimizing potential side effects. Balvert *et al.* (2019) conducted personalized drug prescription based on information from cancer cell lines by selecting the prediction model with the best performance for each drug. In a recent review paper, Romagnoli *et al.* (2017) discussed the information needed for making clinical recommendations about potential drug–drug interactions. More closely related to our problem setting, Rao *et al.* (2020) proposed a medicine recommendation system based on patient reviews. However, their method is focused on finding the best drug for a given condition without personalizing the recommendation based on individual patients’ information; i.e. their recommendation is at the condition level while our method moves one step further to the patient level.

The second class of methods aims to recommend a list of candidate drugs by identifying patients’ symptoms. This class of methods is closely related to the recommendation system widely applied in E-commerce, which addresses the information overload issue by identifying customers with similar behavior patterns and then recommending similar items for potential purchase. Such recommender systems often focus on identifying patient or drug cohorts via clustering and utilize patient–drug interactions, and thus do not apply to our problem setting, due to the well-known cold-start issue (Bobadilla *et al.* 2013). Sun *et al.* (2016) utilized electronic medical records to assign patients into patient cohorts. Then, the typical treatment regimen would be estimated for each patient cohort and recommended for incoming patients classified to the same patient cohort. Zhang *et al.* (2015) proposed a hybrid framework to recommend drugs by matching a new patient’s information with a certain diagnosis. Then, a drug cluster would be recommended based on a pretrained symptom-drug classifier. Readers can refer to Stark *et al.* (2019) for a more detailed overview of this class of medicine recommendation systems.

Due to the differences in problem setting and information needed, the prediction-based methods (i.e. the first class of methods rather than the second class) will be compared with our proposed method in our data analyses.

### 2.2 Multitask learning

When dealing with multiple related or similar modeling tasks, the model performance can be improved by making the tasks “learn from each other.” Such methods are usually referred to as multitask learning. Most of existing multitask learning methods were proposed by researchers in areas such as data mining, statistics, and computer science. Those methods can be classified into two large groups. One group works by assuming a certain structure on the parameters across different tasks to enforce the commonality or similarity. Among the earliest attempts, Liao and Carin (2005) and Silver *et al.* (2008) proposed to use a feed-forward neural network (NN) to learn the best feature transformation by determining the structure of a hidden layer. Obozinski *et al.* (2006, 2010) are among the first to conduct feature selection in the multitask learning setting. Another group of methods focuses on learning the relationships among related tasks. Xue *et al.* (2007) proposed to use a Dirichlet process prior to conduct task clustering. Recently, researchers started to apply multitask learning techniques to healthcare-related problems. More related to our study, Lin *et al.* (2017) proposed a BMul approach for risk profiling in a chronic care setting. For a more detailed review of multitask learning, readers can refer to Zhang and Yang (2022). To the best of our knowledge, our work is the first multitask learning model proposed for ordinal response variables. Furthermore, we are the first to apply the proposed framework to build a medicine recommendation system.

## 3 Data description and system design

We collect patient review data from WebMD.com, a widely used platform for sharing information pertaining to human health and well-being. For a given drug, from each review we have information about the star ratings (1–5) on three measures: satisfaction level, effectiveness, and ease of use; we also have patient information (gender, age group, time since treatment) and the actual review text. In order to capture the main characteristics of the drug reflected through the review contents, we use topic modeling to extract a number of relevant topics from the text information; and for each patient, we create a vector of binary variables to indicate whether each selected topic is covered or not in the review. Details about topic modeling will be discussed in Section 5.

**Figure 1.**
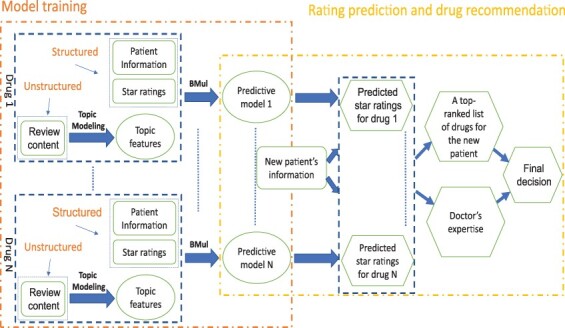
An overview of the design of the proposed medicine recommendation system. We use rectangles to denote inputs, ovals to denote intermediate outputs, and hexagons to denote final outputs

For an incoming patient with a certain health condition, we aim to predict his star ratings on three aspects (satisfaction, effectiveness, and ease of use) of various drugs available for treatment. Using the prediction scores of any aspect, we can generate a ranked list of drugs. Alternatively, an overall preference score, which combines the three aspects, can be used for ranking. To provide final drug recommendations, it is crucial to integrate doctors’ domain knowledge with the predicted rankings. An overview of the design of the proposed medicine recommendation system is presented in Fig. 1. Note that we would like to use the proposed method for new patients whose review contents may not be available. Thus, we make use of the text information collected from existing patients only to extract general drug characteristics. In practice, a questionnaire can be designed and then distributed to new patients during their first visits, to collect information about whether they are concerned with/interested in these characteristics. The information will be supplied along with other patient information such as age and gender to the proposed recommendation system for prediction. In case such information is missing, we can impute the corresponding values using the most frequently occurring values from existing patients for the drug.

For illustrative purposes, we focus on two sets of drugs in our analyses, one for treating depression and the other for high blood pressure, for which the descriptive statistics are given in Tables 1 and 2, respectively. In the data sets, we have information about patients’ gender, age group, and treatment group besides their ratings. The age group is coded as 1 (age 3–6), 2 (age 7–12), 3 (age 13–18), 4 (age 19–24), 5 (age 25–34), 6 (age 35–44), 7 (age 45–54), 8 (age 55–64), 9 (age 65–74), and 10 (age 75 or over). The treatment group is coded as 1 (on treatment for <1 month), 2 (on treatment for 1–6 months), 3 (on treatment for 6–12 months), 4 (on treatment for 1–2 years), 5 (on treatment for 2–10 years), and 6 (on treatment for 10 years or more). An interesting observation from the tables is that, for all the drugs, the mean ratings for satisfaction level are lower than both means for ease of use and effectiveness, suggesting that ease of use and effectiveness are not the only factors that affect patients’ satisfaction. Information such as side effects may be helpful, which can be reflected in some patients’ text reviews.

**Table 1.** Descriptive statistics for patient ratings and characteristics about depression-related drugs.^a^

Drug name	*n*	Satisfaction	Ease of use	Effectiveness	Gender	Age	Treatment	No. of words
1. Amitriptyline	110	2.96(1.64)	4.11(1.22)	3.28(1.52)	0.25(0.43)	6.76(1.28)	3.01(1.39)	50.33(54.22)
2. Bupropion HCl	150	2.95(1.46)	4.26(0.99)	3.18(1.38)	0.27(0.44)	6.57(1.61)	3.31(1.31)	85.03(70.75)
3. Celexa	862	3.32(1.54)	4.32(1.12)	3.60(1.41)	0.19(0.40)	6.29(1.55)	3.11(1.26)	67.19(59.51)
4. Citalopram	606	3.33(1.42)	4.31(1.14)	3.57(1.31)	0.31(0.46)	6.51(1.63)	3.02(1.10)	54.95(48.13)
5. Effexor XR	1300	3.09(1.55)	4.06(1.27)	3.65(1.34)	0.16(0.36)	6.40(1.43)	3.81(1.23)	80.04(70.19)
6. Lexapro	1296	3.33(1.52)	4.36(1.11)	3.68(1.39)	0.20(0.40)	6.20(1.58)	3.12(1.12)	63.31(58.80)
7. Nortriptyline	47	3.06(1.59)	4.13(1.26)	3.44(1.51)	0.23(0.43)	7.00(1.62)	3.03(1.48)	75.66(80.11)
8. Prozac	670	3.21(1.60)	4.26(1.20)	3.45(1.49)	0.17(0.37)	6.24(1.69)	3.54(1.52)	63.99(58.00)
9. Ritalin	28	3.86(1.21)	4.29(0.94)	4.00(1.28)	0.39(0.50)	6.79(1.57)	2.75(1.22)	75.00(71.57)
10. Trazodone	137	3.23(1.61)	3.99(1.28)	3.36(1.49)	0.28(0.45)	6.97(1.52)	3.56(1.53)	40.77(41.89)
11. Wellbutrin XL	773	3.05(1.64)	4.22(1.17)	3.31(1.53)	0.17(0.38)	6.13(1.48)	3.15(1.29)	77.58(64.25)

a For each task/measure, we report the mean and standard deviation of the star ratings (in parentheses). For gender, 0 = Female, 1 = Male.

**Table 2.** Descriptive statistics for patient ratings and characteristics about high blood pressure-related drugs.^a^

Drug name	*n*	Satisfaction	Ease of use	Effectiveness	Gender	Age	Treatment	No. of words
1. Atenolol	600	3.19(1.50)	4.27(1.09)	3.66(1.30)	0.37(0.48)	7.53(1.39)	4.03(1.39)	44.19(45.93)
2. Bystolic	535	2.53(1.47)	3.98(1.28)	3.21(1.43)	0.39(0.49)	7.59(1.29)	2.48(0.78)	58.55(53.65)
3. Hydrochlorothiazide	508	2.65(1.54)	3.98(1.33)	3.24(1.43)	0.29(0.46)	7.41(1.45)	3.26(1.29)	44.96(44.54)
4. Lisinopril	2758	2.50(1.53)	3.96(1.31)	3.32(1.39)	0.38(0.49)	7.59(1.29)	3.16(1.27)	43.84(43.95)
5. Losartan	381	2.41(1.47)	3.82(1.35)	2.96(1.40)	0.35(0.48)	8.17(1.21)	2.86(1.10)	46.47(42.29)
6. Toprol XL	298	2.93(1.54)	4.06(1.25)	3.47(1.40)	0.38(0.49)	7.54(1.23)	3.97(1.21)	52.39(53.72)

a For each task/measure, we report the mean and standard deviation of the star ratings (in parentheses). For gender, 0 = Female, 1 = Male.

For each drug, we examine the correlation structure among the three ratings. The correlations for the 11 depression-related drugs and the 6 high blood pressure-related drugs are shown in Table 3. We can see that the three measures are positively correlated for all the drugs, with a majority of the correlations above 0.5. Also, it is interesting to see that the highest correlations are between satisfaction and effectiveness, ∼0.9 for depression-related drugs and ∼0.75 for high blood pressure-related drugs. These findings show that the three tasks are indeed related, suggesting that a multitask learning framework might be helpful in improving the predictive performance.

**Table 3.** Correlations among satisfaction, effectiveness, and ease of use for drugs related to depression and drugs related to high blood pressure.^a^

	Depression								
	Drug 1	Drug 2	Drug 3	Drug 4	Drug 5	Drug 6	Drug 7	Drug 8	Drug 9
Satisfaction versus ease of use	0.53	0.32	0.53	0.56	0.56	0.52	0.58	0.52	0.56
Satisfaction versus effectiveness	0.90	0.89	0.86	0.88	0.77	0.84	0.81	0.89	0.94
Effectiveness versus ease of use	0.51	0.36	0.56	0.58	0.54	0.52	0.66	0.53	0.50

	Depression		High blood pressure					
	Drug 10	Drug 11	Drug 1	Drug 2	Drug 3	Drug 4	Drug 5	Drug 6
Satisfaction versus ease of use	0.58	0.48	0.50	0.44	0.46	0.47	0.52	0.56
Satisfaction versus effectiveness	0.87	0.88	0.75	0.75	0.73	0.64	0.76	0.79
effectiveness versus ease of use	0.55	0.48	0.56	0.43	0.43	0.55	0.43	0.58

a *P*-values are <.01 for all the correlation tests.

## 4 Materials and methods

### 4.1 Ordinal regression and the latent variable view

We first give a brief overview of the base model we use for predicting star ratings. This serves as a major building block for the BMul model that will be introduced in the next subsection. Note that, throughout the article, the prediction is done separately for each drug. For a specific drug, let $y_{ij}\in \{1,2,\ldots ,R=5\}$ be the rating of the *i*th reviewer (patient) for measure *j*, i=1,…,n, j=1,…,J=3, where *n* is the total number of reviewers available for that drug. A generalized linear regression model for an ordinal response variable with a link function *g* assumes the following form: where $x_{ij}$ is a column vector of *p* covariates; for each measure *j*, $\alpha_{r,j}$ is the intercept that depends on *r* and satisfies $\alpha_{1,j}<\alpha_{2,j}<\ldots <\alpha_{R-1,j}$, and $b_{j}={\left(b_{1j},\ldots ,b_{pj}\right)}^{T}$ are the regression coefficients that are assumed common for all *r* values. In particular, for reviewer *i*, we set the covariates to be: gender (0 = Female, 1 = Male), age group (recoded to 1–9), treatment length, the quadratic terms of age group and treatment length, and indicators for which topics are covered in the review.


(1) $$g\left\{P(y_{ij}\leq r)\right\}=\alpha_{r,j}+x_{ij}^{T}b_{j},\;r\in \{1,\ldots ,R-1\},$$

There exists a latent variable view for the model (1), which allows us to easily incorporate it into a Bayesian framework (Albert and Chib 1993). Let $y_{ij}^{*}$ be an underlying latent variable with $P(y_{ij}^{*}\leq t|x_{ij},b_{j})=G(t+x_{ij}^{T}b_{j})$, where *G* is the cumulative density function (CDF) of a user-specified distribution. Assume the observed response $y_{ij}$ depends on the unobserved variable $y_{ij}^{*}$ through the following relationship, $y_{ij}=r\;\text{if}\;\alpha_{r-1,j}<y_{ij}^{*}\leq \alpha_{r,j},$ where $-\infty =\alpha_{0,j}<\alpha_{1,j}<\ldots <\alpha_{R-1,j}<\alpha_{R,j}=\infty$ are cutoff values for segmenting $y_{ij}^{*}$. Then leading to model (1) if we set $g=G^{-1}$.


$$P(y_{ij}\leq r|x_{ij},b_{j})=P(y_{ij}^{*}\leq \alpha_{r,j}|x_{ij},b_{j})=G(\alpha_{r,j}+x_{ij}^{T}b_{j}),$$

If *G* is the CDF of a standard logistic distribution, then the resulting model is an ordinal logistic regression model. For an efficient and easy implementation of the Bayesian method introduced in subsequent subsections, we set *G* to be the CDF of a standard normal distribution Φ, which is equivalent to assuming $y_{ij}^{*}=x_{ij}^{T}\beta_{j}+\epsilon_{ij}$, with $\beta_{j}\equiv -b_{j}$ and $\epsilon_{ij}∼N(0,1)$ so that


(2) $$P(y_{ij}\leq r|x_{ij},\beta_{j})=P(y_{ij}^{*}\leq \alpha_{r,j}|x_{ij},\beta_{j})=\Phi (\alpha_{r,j}-x_{ij}^{T}\beta_{j}).$$

We will refer to the above model as the ordinal probit model (OP), one of the baseline models used for performance comparison.

### 4.2 The BMul model

To account for the fact that the star ratings on different measures are for the same drug and from the same reviewer, we assume that the prior of $\beta_{k.}={\left(\beta_{k1},\ldots ,\beta_{kJ}\right)}^{T}$ for the *k*th covariate follows a multivariate normal distribution:


$${\left(\beta_{k1},,\ldots ,\beta_{kJ}\right)}^{T}∼N(0,\Sigma_{k}),\;\;\;\;k=1,\ldots ,p.$$

We further assign a hyperprior distribution to each $\Sigma_{k}$: $\Sigma_{k}∼W^{-1}(\Psi_{k},\nu )$, which is an inverse Wishart distribution with hyperparameters $\Psi_{k}$ (a J×J scale matrix) and ν>J−1 (the degrees of freedom). In this way, although the parameters of the same covariate for *J* measures are different, they are set to learn from each other through the dependent multivariate normal prior and the corresponding hierarchical Bayes structure. Letting $\beta ={\left(\beta_{11},\ldots ,\beta_{1J},\ldots ,\beta_{p1},\ldots ,\beta_{pJ}\right)}^{T}$, the prior for β can be rewritten as β∼N(0,Σ), where $\Sigma =\text{diag}(\Sigma_{1},\ldots ,\Sigma_{p})$. We assume the prior distributions of $\alpha_{j}={\left(\alpha_{1,j},\ldots ,\alpha_{R-1,j}\right)}^{T}$ are $\alpha_{1,j}∼N(0,10)$, $\alpha_{r,j}|\alpha_{r-1,j}∼N(0,10)$ left truncated at $\alpha_{r-1,j}$, r=2,…,R−1. Let $\alpha ={\left(\alpha_{1,1},\ldots ,\alpha_{R-1,1},\ldots ,\alpha_{1,J},\ldots ,\alpha_{R-1,J}\right)}^{T}$. Then the joint posterior distribution of all parameters and latent variables can be written as: where $y={\left(y_{11},\ldots ,y_{1J},\ldots ,y_{n1},\ldots ,y_{nJ}\right)}^{T}$, is a vector of size *nJ*, $y^{*}={\left(y_{11}^{*},\ldots ,y_{1J}^{*},\ldots ,y_{n1}^{*},\ldots ,y_{nJ}^{*}\right)}^{T}$, and *X* is the corresponding covariate matrix shown below, whose dimension is pJ×nJ, with $x_{\mathrm{ijk}}$ being the *k*th element of $x_{ij}$ (i.e. the value of covariate *k* for reviewer *i* and measure *j*):


$$\begin{array}{c} p(\alpha ,\beta ,\Sigma ,y^{*}|y,X)\\ \propto \prod_{r,j}\prod_{i:y_{ij}=r}I(\alpha_{r-1,j}<y_{ij}^{*}<\alpha_{r,j})\cdot \prod_{i,j}p(y_{ij}^{*}|x_{ij},\beta_{j})\\ \cdot \prod_{j}\left[p(\alpha_{1,j})\prod_{r=2}^{R-1}p(\alpha_{r,j}|\alpha_{r-1,j})\right]\cdot p(\beta |\Sigma )\cdot p(\Sigma ), \end{array}$$

$$\begin{array}{c} X=\left(\begin{array}{ccccccccc} x_{111} & 0 & \cdots & 0 & \cdots & x_{n11} & 0 & \cdots & 0\\ 0 & x_{121} & \cdots & 0 & \cdots & 0 & x_{n21} & \cdots & 0\\ \vdots & \vdots & \ddots & \vdots & \cdots & \vdots & \vdots & \ddots & \vdots \\ 0 & 0 & \cdots & x_{1J1} & \cdots & 0 & 0 & \cdots & x_{nJ1}\\ \vdots & \vdots & \vdots & \vdots & \vdots & \vdots & \vdots & \vdots & \vdots \\ x_{11p} & 0 & \cdots & 0 & \cdots & x_{n1p} & 0 & \cdots & 0\\ 0 & x_{12p} & \cdots & 0 & \cdots & 0 & x_{n2p} & \cdots & 0\\ \vdots & \vdots & \ddots & \vdots & \cdots & \vdots & \vdots & \ddots & \vdots \\ 0 & 0 & \cdots & x_{1Jp} & \cdots & 0 & 0 & \cdots & x_{\mathrm{nJp}} \end{array}\right). \end{array}$$

We note that in our application, for any patient *i*, the covariate vectors $x_{ij}$’s for different star-rating measures are the same, as the information available for each measure is the same. That is, we have $x_{\mathrm{ijk}}\equiv x_{ij^{\prime }k}\;\forall j,j^{\prime }$ such that each J×J submatrix of *X* has identical diagonal components. However, throughout the article, for a general model set-up, we use $x_{ij}$ rather than $x_{i}$ to allow the covariate vectors for different measures to be different. Later in the variable selection stage, we allow different variables to be selected for different measures and using $x_{ij}$ would make this explicit.

To sample from the posterior distribution, we use a Gibbs sampler, which iteratively samples the parameters from the following conditional posteriors:


$$\begin{array}{c} p(\alpha_{j}|y^{*},y,X,\beta ,\Sigma )=p(\alpha_{j}|y^{*},y)\\ \propto p(\alpha_{1,j})\prod_{r=2}^{R-1}p(\alpha_{r,j}|\alpha_{r-1,j})\prod_{r=1}^{R}\prod_{i:y_{ij}=r}I(\alpha_{r-1,j}<y_{ij}^{*}<\alpha_{r,j}),\\ \left(\beta |y^{*},y,X,\alpha ,\Sigma )=(\beta |y^{*},X,\Sigma \right)\\ ∼N\left({\left(XX^{T}+\Sigma^{-1}\right)}^{-1}Xy^{*},{\left(XX^{T}+\Sigma^{-1}\right)}^{-1}\right),\\ (\Sigma_{k}|y^{*},y,X,\alpha ,\beta )=(\Sigma_{k}|\beta_{k.})∼W^{-1}(\Psi_{k}+\beta_{k.}\beta_{k.}^{T},\nu +1),\\ \left(y_{ij}^{*}|y_{-(ij)}^{*},y,X,\alpha ,\beta )=(y_{ij}^{*}|y_{ij}=r,\alpha_{j},\beta_{j}\right)\\ ∼N(x_{ij}^{T}\beta_{j},1)\;\text{truncated between}\;\alpha_{r-1,j}\;\text{and}\;\alpha_{r,j}. \end{array}$$

### 4.3 A Simplified BMul model

One simplification of the BMul model is to set $\alpha_{j}$ to be the same for different *j* in (1). This assumption is plausible in our application because the three measures are for the same drug and on the same scale (from 1 to 5), and so the intercepts might not vary much across these measures. Under this assumption, the joint posterior distribution can be reduced to


$$\begin{array}{c} p(\alpha ,\beta ,\Sigma ,y^{*}|y,X)\propto \prod_{r,j}\prod_{i:y_{ij}=r}I(\alpha_{r-1}<y_{ij}^{*}<\alpha_{r})\cdot \prod_{i,j}p(y_{ij}^{*}|x_{ij},\beta_{j})\\ \left[p(\alpha_{1})\prod_{r=2}^{R-1}p(\alpha_{r}|\alpha_{r-1})\right]\cdot p(\beta |\Sigma )\cdot p(\Sigma ). \end{array}$$

To examine how this parsimony assumption would affect the performance, we conduct a small simulation study to compare the two versions (same intercepts versus different intercepts across *j*). We use data from the first three depression-related drugs (i.e. Drugs 1–3 in Table 1) as examples and for each drug, we summarize results in Table 4 based on 100 repetitions, where each repetition randomly selects 50 patients for training and then uses the remaining for testing (for more detail, see the beginning of Section 5). Columns 2–5 show the prediction accuracy and AUC (area under the receiver operating characteristic curve) in percentages for running a 5-class model (i.e. each $y_{ij}$ has five levels, one star to five stars), and Columns 6−9 for a 2-class model (i.e. each $y_{ij}$ has two levels, <3 versus ≥3 stars, which can be interpreted as “not recommend” versus “recommend”). For each model, the first two columns show results for assuming $\alpha_{j}\equiv \alpha$ and the latter two for $\alpha_{j}\neq \alpha$. Unless specified otherwise, the accuracy and AUC values are calculated from the test data by pooling all three measures throughout the article; for both 5-class and 2-class BMul models, they are computed using the predicted probability of “recommend,” and the cutoff for computing the accuracy is the default value 0.5. The rows show results for different drugs. We find that the performance for assuming the same intercepts across different *j* leads to comparable results, both in terms of accuracy and AUC. Therefore, the simplified BMul with constant intercepts will be used for the rest of the article. In addition, since the 5-class BMul performs slightly better than the 2-class BMul, the 5-class label is to be used for the training stage for the rest of the article.

**Table 4.** A comparison of the simplified model assuming the same intercepts across different *j* and the original model using resampled data for the first three depression-related drugs.^a^

	Five-class BMul				Two-class BMul			
	$\alpha_{j}\equiv \alpha$		$\alpha_{j}\neq \alpha$		$\alpha_{j}\equiv \alpha$		$\alpha_{j}\neq \alpha$	
	Accuracy	AUC	Accuracy	AUC	Accuracy	AUC	Accuracy	AUC
Drug 1	76.2	72.0	76.1	71.8	75.3	69.6	75.4	69.5
Drug 2	73.1	70.8	73.3	71.2	72.7	70.8	72.7	70.6
Drug 3	79.4	69.9	78.6	69.0	78.9	68.2	78.9	68.4

a Both the accuracy and AUC are reported in percentages.

### 4.4 BMul with LASSO for variable selection

**Figure 2.**
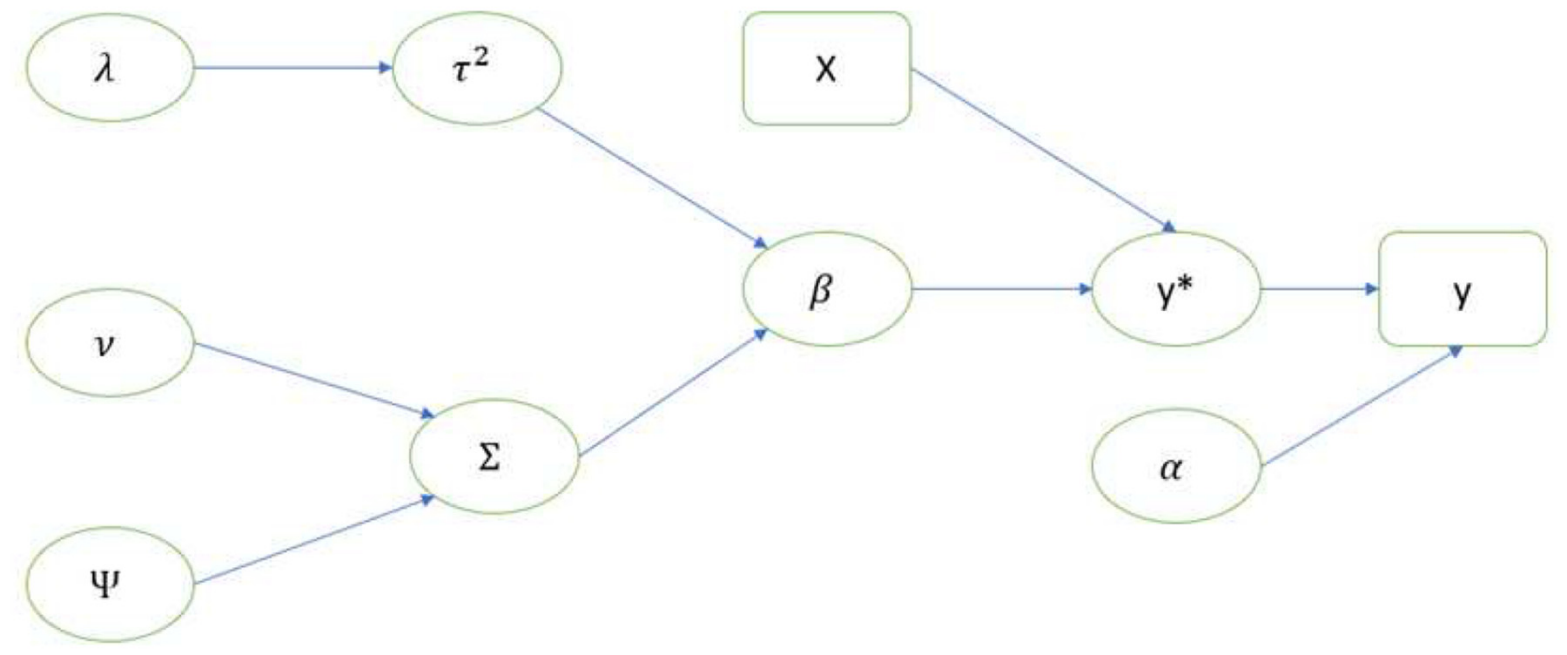
A diagram for the proposed Bayesian multitask learning with LASSO for variable selection

In many applications, not all the covariates are useful for the prediction task. In our context, we conjecture that at least some topic indicator variables generated from the review contents might be irrelevant. Further, hospitals and other medical services can have much more information about individual patients. Thus, we introduce a modified BMul model with an added hierarchical structure for the purpose of variable selection, which helps to select important variables among a potentially large pool of candidate variables. To encourage the sparsity of β, we introduce a shrinkage parameter $\tau_{k}^{2}$ for $\Sigma_{k}$, the covariance matrix of $\beta_{k.}$. That is, the prior on $\beta_{k.}={\left(\beta_{k1},\ldots ,\beta_{kJ}\right)}^{T}$ is set to $\beta_{k.}∼N(0,\Xi_{k})$, where $\Xi_{k}\equiv \tau_{k}^{2}\Sigma_{k}$, $\tau_{k}^{2}∼\lambda^{2}\;\text{exp}(-\lambda^{2}\tau_{k}^{2})/2$, and λ is a hyperparameter that can be estimated using an empirical Bayesian approach. Such a hierarchical structure is motivated by the seminal work for Bayesian LASSO proposed by Park and Casella (2008). A diagram for our modified Bayesian model with LASSO is shown in Fig. 2, where the rectangular-shaped items denote observed variables and the oval-shaped items denote either parameters or hidden variables.

Letting $\tau^{2}={\left(\tau_{1}^{2},\ldots ,\tau_{p}^{2}\right)}^{T}$, the conditional posterior distributions for $\beta_{k.},\tau_{k}^{2}$ and $\Sigma_{k}$ now become where *IG* stands for an inverse-Gaussian distribution, $\Xi =\text{diag}(\Xi_{1},\ldots ,\Xi_{p})$, and the conditional posterior distributions for α and $y^{*}$ remain unchanged. The estimates for the hyperparameter λ can be updated at every iteration using the method of maximum marginal likelihood. In particular, at iteration *t*, the hyperparameter $\lambda^{\left(t\right)}$ can be updated by $\lambda^{\left(t\right)}=\sqrt{(2p)/\{\sum_{k=1}^{p}E_{\lambda^{\left(t-1\right)}}(\tau_{k}^{2})\}.}$


$$\begin{array}{c} (\frac{1}{\tau_{k}^{2}}|\tau_{-k}^{2},y^{*},y,\alpha ,\beta )=(\frac{1}{\tau_{k}^{2}}|\beta_{k.})∼IG(\mu^{\prime },\lambda^{\prime }),\;\text{where}\;\mu^{\prime }=\sqrt{\frac{\lambda^{2}}{\beta_{k.}^{T}\beta_{k.}}},\lambda^{\prime }=\lambda^{2}\\ \left(\beta |y^{*},y,X,\alpha ,\Sigma ,\tau^{2})=(\beta |y^{*},X,\Sigma ,\tau^{2}\right)\\ ∼N({\left(XX^{T}+\Xi^{-1}\right)}^{-1}Xy^{*},{\left(XX^{T}+\Xi^{-1}\right)}^{-1}),\\ (\Sigma_{k}|y^{*},y,X,\alpha ,\beta )=(\Sigma_{k}|\beta_{k.},\tau_{k}^{2})∼W^{-1}(\Psi_{k}+\tau_{k}^{-2}\beta_{k.}\beta_{k.}^{T},\nu +1), \end{array}$$

### 4.5 Algorithm implementation and model comparison

Since we set all the priors to be conjugate, as shown in the previous subsections, the conditional posterior distribution of each parameter follows a known distribution. As a result, sampling from the posterior can be easily achieved using Gibbs sampling (Geman and Geman 1984). For all the analyses in the article, we use Geweke’s Diagnostics (implemented in R package “coda,” Version: 0.19-4) to check the convergence and use the sample means of the second halves of the chains as the estimates.

**Figure 3.**
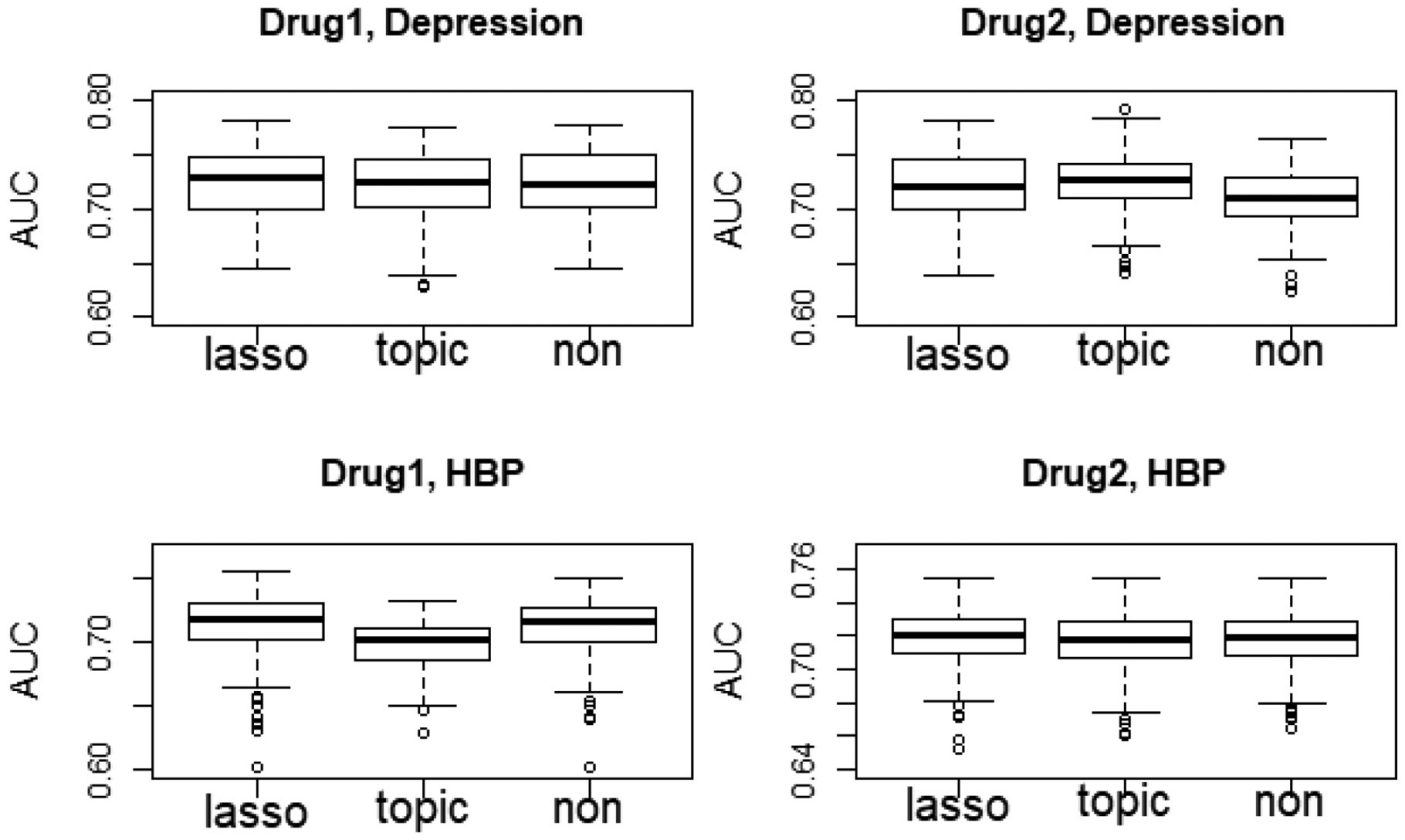
Comparison of BMul methods with and without the variable selection component using resampled data for the first two depression-related drugs and the first two high blood pressure-related drugs. Here, “topic”/“non” stands for BMul with/without using topic-related variables, neither conducting variable selection; and “lasso” stands for BMul with LASSO for variable selection

To examine how the (modified) BMul works in practice, we use the first two depression-related drugs and the first two high blood pressure-related drugs as examples, run 100 repetitions for each drug, and compare the AUC values from the BMul methods with and without the variable selection component (i.e. BMul_lasso, BMul_topic, and BMul_non). Here, BMul_lasso stands for BMul with LASSO for variable selection, BMul_topic for BMul using all the topic-related variables, and BMul_non for BMul excluding all the topic-related variables. Neither BMul_topic nor BMul_non employs variable selection. The AUC values are calculated using the probability of “recommend” as in Table 4, whose boxplots are shown in Fig. 3 by drug.

In situations when some or all variables from topic modeling contain useful information for predicting the star ratings, we expect BMul_topic to perform better than BMul_non; otherwise, BMul_topic would perform comparably to or even worse than BMul_non, due to inclusion of many irrelevant variables. Figure 3 shows the four drugs fit in the different situations, where BMul_lasso mimics the better of the two when BMul_topic and BMul_non perform differently. For depression drug 2, BMul_topic performs better than BMul_non. We can see that BMul_lasso is able to select useful topics and thus produces AUC closer to that of BMul_topic. On the other hand, BMul_topic performs worse than BMul_non for high blood pressure drug 1. In this case, BMul_lasso is able to remove the majority of the topics and thus produces AUC similar to that of BMul_non. For depression drug 1 and high blood pressure drug 2, the performance of BMul_topic and BMul_non is similar, so is that of BMul_lasso. These results suggest that BMul_lasso serves the purpose of variable selection and is effective in selecting the relevant variables (if any) and excluding irrelevant ones. Thus, BMul_lasso is a better choice as we typically do not know whether topic modeling can help or not in creating variables to predict star ratings for a specific drug.

To test the significance of differences between the methods with and without feature selection, we conduct a two-way ANOVA analysis for the logit-transformed AUC with two factors (i.e. method and drug), in which BMul_lasso serves as the baseline method. The negative coefficients for BMul_non and BMul_topic in Table 5 show that BMul_lasso outperforms both. Further, the differences are statistically significant at the 5% significance level.

**Table 5.** Testing the significance of differences in AUC between the BMul methods with and without feature selection: a two-way ANOVA model for the logit-transformed AUC with two factors method and drug, where BMul_lasso and drug 1 are coded as the baseline for each factor, respectively.^a^

	Estimate (SE)	Significant?		Estimate (SE)	Significant?
Intercept	0.975 (0.010)	***	Drug 2	−0.020 (0.011)	**
BMul_non	−0.021 (0.009)	**	Drug 3	0.069 (0.011)	***
BMul_topic	−0.024 (0.009)	***	Drug 4	−0.032 (0.011)	***

a Note that “*,” “**,” and “***” represent that the result is statistically significant at the 10%, 5%, and 1% level, respectively.

We refer to BMul with LASSO for variable selection as BMull hereafter. For the performance comparison in Section 5, we will use the BMull version.

### 4.6 Method limitations

The proposed methodology offers an effective means of predicting patients’ star ratings on three different aspects of a drug simultaneously. In the field of recommender research, there are many proposed methods for combining criteria-specific ratings into an overall rating. Simple approaches include weighted averages or additive methods based on utility functions (Lakiotaki *et al.* 2008). More advanced techniques use probability distributions to model user preferences across criteria, such as probabilistic latent semantic analysis (Zhang *et al.* 2009). Multiple-criteria decision analysis can also be used to consolidate the different scores (Lakiotaki *et al.* 2011). However, all these methods require data containing user-assigned overall ratings, which serve as supervision to guide the aggregation process. Such overall ratings are unavailable in our WebMD.com datasets. Furthermore, the recommendation of drugs should consider both doctors’ domain knowledge and patients’ ratings, usually incorporated through a utility function, which is not available in our study.

In real-world scenarios, one can always take the average of predicted multicriteria ratings but this may often be insufficient. It is important to highlight that doctors have the ability to leverage patient-specific information, along with their domain knowledge, to make recommendations based on individual patient preferences across the three criteria. This personalized approach can significantly enhance the accuracy and relevance of drug recommendations. Unfortunately, due to the unavailability of sensitive and personalized data required for such analysis, coupled with the absence of overall rating data and a utility function, we were unable to conduct an experiment to demonstrate the real-world effectiveness of our methodology. We recognize the value of incorporating patient-specific information and data into the recommendation process, but it is beyond the scope of our current study.

Finally, we emphasize that the proposed methodology is intended to be a useful source of information for doctors, rather than being directly used by patients. Caution should be exercised as we are limited by publicly available features and data quality. However, the transparency and explainability of our method enable doctors not only to resort to the predicted ratings but also to understand how the ratings are generated. Further, since it is a Bayesian method, the prediction interval is a natural by-product of posterior sampling, which can help gauge the prediction uncertainty and raise better awareness about the limitations of the output.

## 5 Data analysis results

We apply the proposed BMull (Bayesian multitask learning with LASSO) and a list of competitors containing popular classification algorithms to predict patient ratings for different drugs. The list includes the OP, support vector machine (SVM), random forests (RF), NN and AdaBoost (Ada). For each of these existing methods, we consider two versions, with and without using the topic information (_topic versus _non). We report results based on averages over 100 repetitions for each drug. For each repetition, we randomly sample 50 patients and use the corresponding 150 observations (three measures for each patient) to form a training set, and all the remaining observations to form a test set. The accuracy and AUC measures are calculated using the test set. For all methods, the 5-class label is used in the training stage, and the probability of “recommend” (i.e. three stars or above) is used for computing the accuracy (using the cutoff value 0.5) and AUC values in the testing stage. In what follows, we first describe the data processing steps and then report the analysis results for performance evaluation.

### 5.1 Data preprocessing

As described earlier, in addition to the numeric values describing each patient’s demographics, we have the actual review content from each patient. We use topic modeling to extract the commonly mentioned features about each drug and use them as part of our knowledge base when recommending a drug to new patients. For some of the drugs, the number of reviews is limited and topic modeling may not produce satisfactory results. Thus, we augment our review samples by including reviews of the same drugs from a Kaggle data set (available at https://www.kaggle.com/jessicali9530/kuc-hackathon-winter-2018). Using the augmented review samples, we conduct topic modeling (Blei *et al.* 2003) for each drug by setting the number of topics to be 4, 7, 10, 13, 16, and 19, and use the coherence score for each model as the criterion to select the best topic number. Here, each topic is typically represented as a set of important (or top ranked) words, and the coherence score of a model measures semantically how similar these words within each topic are. Therefore, a higher coherence score means tighter clusters for a model. Further technical details about topic modeling and the preprocessing steps are included in the Supplementary Material.

As the output of topic modeling, for each review, we obtain a probability vector $\pi =(\pi_{1},\ldots ,\pi_{K})$, where *K* is the total number of topics selected based on the coherence score, and $\pi_{k}$ is a probability measuring how likely this review is related to topic *k*, satisfying $\sum_{k=1}^{K}\pi_{k}=1$. Then, we use 1/K as the cutoff to create the corresponding topic indicator. For example, if θ=(0.1,0.02,0.7,0.18) for a review, then the cutoff is 0.25 and the topic indicator vector is (0,0,1,0). That is, this review is related to topic 3. Note that the indicator vector can have multiple elements being 1, so a review can be related to multiple topics. For each review, we further introduce the idea of the dominant topic, defined as the topic with the highest probability. In our previous example, the probability vector is θ=(0.1,0.02,0.7,0.18) for topics 0–3, respectively. The highest probability 0.7, corresponds to topic 2. Thus, the dominant topic is 2 for this review.

In addition to the topic indicators, we also extracted the sentiment score (ranging from −1 to 1) for each review, using the textblob function in python. A positive/negative sign of the score reflects a positive/negative sentiment, while the value reflects the magnitude of the sentiment. To incorporate this information into the analysis, we created a variable called prior_sentiment. For a review, its prior_sentiment is defined as the average sentiment score for the other reviews that occur prior to it and have the same dominant topic. Obviously, the first review for each topic will not have any prior reviews. So, we set the prior_sentiment to 0 for such reviews. This sentiment variable and the topic indicators are included in the model for the topic version of each method.

### 5.2 Comparison of predictive performance

We first show accuracy and AUC results for the first two depression-related drugs and the first two high blood pressure-related drugs in Table 6, where for each evaluation criterion, the best/worst number is highlighted in bold/italic font. The results for other drugs follow similar patterns and so are omitted for brevity.

**Table 6.** Performance comparison using resampled data for depression-related drugs 1 and 2 (Columns 2–5) and for high blood pressure-related drugs 1 and 2 (Columns 6–9).^a^

	Depression				High blood pressure			
Methods	Drug 1		Drug 2		Drug 1		Drug 2	
	Accuracy	AUC	Accuracy	AUC	Accuracy	AUC	Accuracy	AUC
BMull	**76.4**	72.0	**73.8**	**72.0**	79.2	**70.1**	**69.7**	**71.3**
	(2.9)	(3.3)	(2.4)	(2.2)	(1.6)	(2.6)	(1.5)	(1.2)
OP_topic	72.4	67.1	71.7	68.5	*76.1*	64.5	67.2	67.5
	(2.6)	(3.3)	(2.1)	(2.8)	(1.2)	(2.2)	(1.6)	(1.7)
OP_non	76.1	72.1	73.1	70.8	79.0	69.5	69.4	70.7
	(3.7)	(4.6)	(3.3)	(3.8)	(2.0)	(2.8)	(2.0)	(2.4)
SVM_topic	75.2	69.3	**73.8**	69.5	**80.1**	62.6	68.0	69.2
	(2.6)	(3.2)	(2.3)	(2.6)	(1.3)	(2.2)	(1.7)	(1.8)
SVM_non	75.5	70.5	**73.8**	67.5	80.0	66.3	68.4	70.5
	(1.9)	(4.6)	(1.6)	(4.4)	(0.4)	(5.0)	(1.4)	(2.6)
RF_topic	76.1	70.4	72.6	71.2	79.0	67.4	69.3	70.7
	(2.2)	(4.0)	(1.5)	(5.1)	(0.5)	(5.6)	(1.8)	(2.6)
RF_non	75.1	**72.2**	71.3	69.9	78.8	68.9	69.2	71.0
	(2.5)	(3.0)	(2.0)	(2.5)	(1.1)	(2.8)	(1.3)	(1.3)
NN_topic	*72.1*	*60.7*	*70.7*	*61.0*	78.9	*57.3*	*65.4*	*60.9*
	(5.6)	(7.1)	(5.0)	(7.1)	(2.7)	(6.4)	(3.9)	(6.6)
NN_non	74.4	62.4	73.1	62.5	79.2	61.3	66.8	62.4
	(3.5)	(7.1)	(2.4)	(7.7)	(2.4)	(6.7)	(2.9)	(7.5)
Ada_topic	73.7	68.5	71.6	70.0	78.2	67.5	68.5	70.0
	(3.1)	(3.7)	(2.7)	(3.1)	(2.1)	(2.3)	(2.0)	(2.4)
Ada_non	74.1	69.9	71.2	69.6	78.2	69.2	69.2	70.7
	(3.0)	(3.8)	(2.8)	(2.8)	(2.0)	(2.6)	(1.5)	(1.5)

a Numbers in brackets indicate standard deviations.

In terms of both AUC and accuracy, BMull performs the best or close to the best. This indicates that the proposed method can effectively borrow information across tasks and therefore achieve better predictive performance. We observe from Table 6 that topic information is not always helpful in boosting the performance of the existing methods. The same observation is shown in Fig. 3 for the proposed method. For example, for depression-related drug 1, using the topic information leads to worse AUC for all the existing methods (the AUC decreases by 1.2 to 5.0). On the other hand, for depression-related drug 2, the topic information lifts the performance of RF, SVM, and Ada but reduces the performance of OP and NN. Overall, we see that adding the topic information negatively affects the performance of OP the most, which is probably due to the overfitting issue. We further note that conducting variable selection for the existing methods might not change the best numbers in Table 6, as doing so would lead to performance somewhere between the two versions (with and without topic information) for a single drug.

To test the significance of differences between the proposed method BMull and the competing methods, we run a two-way ANOVA model for the logit-transformed performance measure (either accuracy or AUC) with two factors method and drug. The results for accuracy and AUC are reported in Table 7. First, the coefficient estimates for the competing methods are all negative, indicating BMull outperforms the others, regardless of the measure. In terms of accuracy, the differences are statistically significant at the 5% significance level, except for SVM_non whose difference is significant at the 10% level. In terms of AUC, all the differences are statistically significant at the 5% level. In addition, the next best method is not the same for accuracy and AUC.

**Table 7.** Testing the significance of differences in accuracy and AUC between the proposed method BMull and competing methods: a two-way ANOVA model for the logit-transformed accuracy and AUC with two factors method and drug, where BMull and drug 1 are coded as the baseline for each factor, respectively.^a^

	Accuracy		AUC	
Features	Estimate (SE)	Significant?	Estimate (SE)	Significant?
Intercept	1.144 (0.007)	***	0.952 (0.011)	***
OP_non	−0.021 (0.009)	**	−0.031 (0.013)	**
OP_topic	−0.182 (0.009)	***	−0.233 (0.013)	***
SVM_non	−0.015 (0.009)	*	−0.143 (0.013)	***
SVM_topic	−0.019 (0.009)	**	−0.176 (0.013)	***
RF_non	−0.066 (0.009)	***	−0.054 (0.013)	***
RF_topic	−0.035 (0.009)	***	−0.082 (0.013)	***
NN_non	−0.051 (0.009)	***	−0.427 (0.013)	***
NN_topic	−0.138 (0.009)	***	−0.521 (0.013)	***
Ada_non	−0.079 (0.009)	***	−0.079 (0.013)	***
Ada_topic	−0.089 (0.009)	***	−0.119 (0.013)	***

Drug 2	−0.106 (0.005)	***	−0.003 (0.008)	NS
Drug 3	0.228 (0.005)	***	−0.111 (0.008)	***
Drug 4	−0.300 (0.005)	***	−0.011 (0.008)	NS

a Note that “*,” “**,” and “***” represent that the result is statistically significant at the 10%, 5%, and 1% level, respectively; “NS” stands for nonsignificant results.

**Figure 4.**
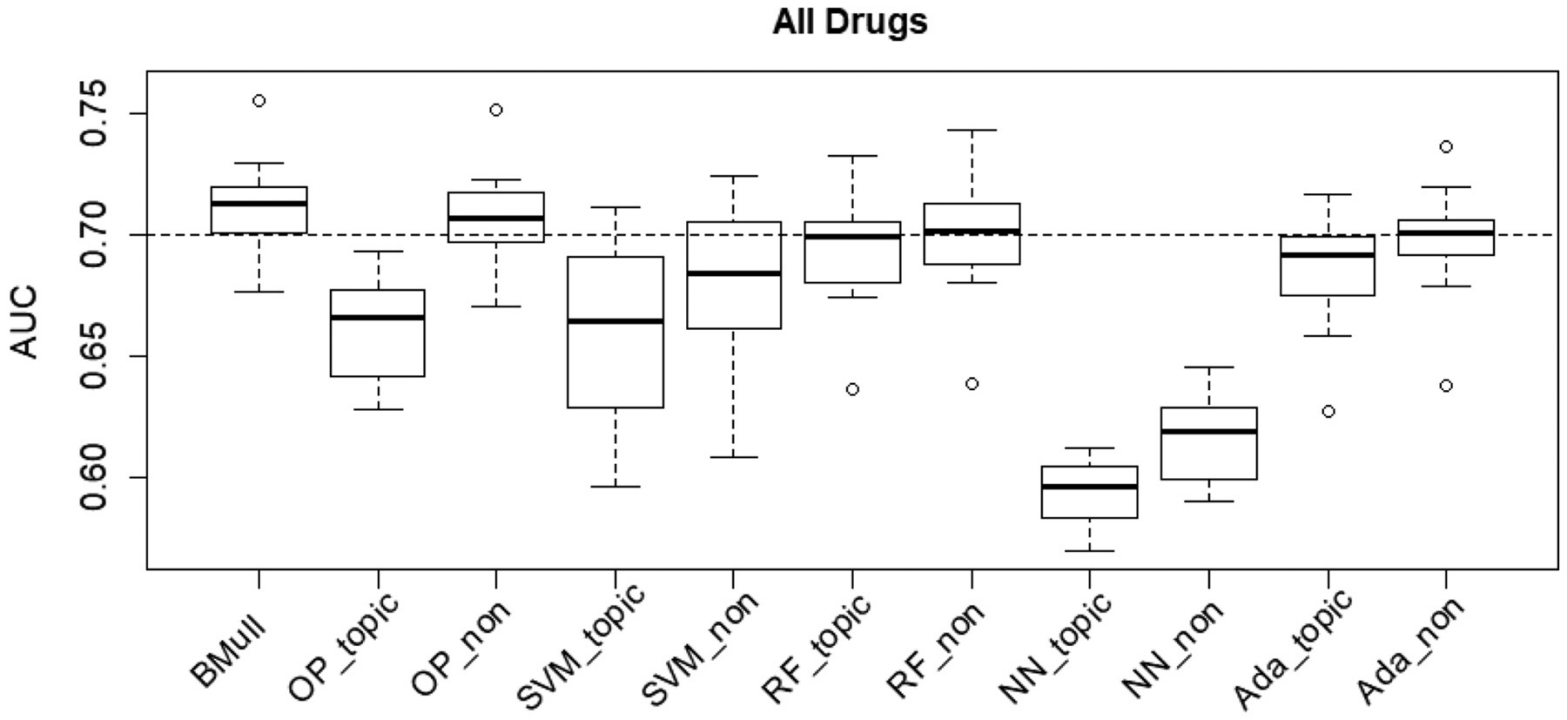
AUC comparison for different methods using resampled data for all depression and high blood pressure-related drugs.

The AUC comparison for all drugs is given in Fig. 4, in which each boxplot shows the AUC distribution for one particular method. The proposed BMull generally achieves better AUC values: its boxplot shows the highest minimum, maximum, first, second, and third quartiles among all, and its first quartile is higher than or comparable to the medians of the others except for that of OP_non. Also, it is more robust to extra noninformative variables meanwhile can efficiently use topic-related information underlying the variables from topic modeling, compared to other methods. In particular, OP has a huge performance decrease from the without-topic version to the with-topic version.

We also evaluate the predictive performance of different methods on each individual task (measure). The results for the first two depression-related drugs are reported in Table 8. Our method always avoids the worst performance and provides the best performance in 5 out of 12 cases. Interestingly, we also observe that the performance for some of the measures is not consistent with the overall performance. For example, our method produces the best overall accuracy for drug 1, as shown in Table 6. However, SVM produces the best accuracy for “ease of use” and our method falls behind a bit. Note that the accuracy for ease of use is quite high and more than 90% for most methods; thus, multitask learning can work by sacrificing the performance of one task a bit while boosting the overall performance.

**Table 8.** Predictive performance for different methods using resampled data for the first two depression-related drugs, evaluated on each of the three individual tasks.

	Drug 1					
Methods	Satisfaction		Ease of use		Effectiveness	
	Accuracy	AUC	Accuracy	AUC	Accuracy	AUC
BMull	61.8	**63.5**	89.7	62.9	75.6	**73.1**
OP_topic	60.8	61.4	*85.3*	60.4	69.5	65.5
OP_non	**63.0**	**63.5**	89.3	**64.0**	75.2	72.6
SVM_topic	61.9	63.0	**90.4**	59.0	73.1	65.1
SVM_non	61.5	63.0	**90.4**	57.9	73.9	70.6
RF_topic	61.0	61.8	**90.4**	55.7	74.7	70.0
RF_non	57.3	61.6	**90.4**	57.6	**76.0**	72.4
NN_topic	60.1	*55.0*	87.6	*52.0*	*69.1*	*58.7*
NN_non	61.2	55.8	90.3	53.3	73.4	61.0
Ada_topic	58.3	60.6	89.7	57.8	73.3	68.3
Ada_non	*57.2*	60.7	89.7	57.4	75.5	72.0

	Drug 2					
Methods	Satisfaction		Ease of use		Effectiveness	
	Accuracy	AUC	Accuracy	AUC	Accuracy	AUC
BMull	**61.3**	**63.4**	**93.3**	63.7	67.4	60.0
OP_topic	60.1	61.8	*90.4*	59.4	65.1	**61.5**
OP_non	60.4	61.1	**93.3**	63.6	66.8	60.4
SVM_topic	59.6	61.4	**93.3**	60.1	69.4	59.1
SVM_non	59.2	59.5	**93.3**	63.0	**69.7**	57.4
RF_topic	58.3	60.0	93.2	**70.9**	66.6	56.5
RF_non	56.4	58.5	**93.3**	67.9	66.3	55.5
NN_topic	60.1	*55.7*	91.4	*55.6*	*64.1*	*53.3*
NN_non	61.2	56.5	92.9	55.7	67.5	55.7
Ada_topic	58.3	60.3	**93.3**	65.6	65.1	56.1
Ada_non	*57.2*	59.6	**93.3**	67.6	66.2	55.5

Bold ones are the highest (best) numbers and the italic ones are the lowest (worst) numbers.

### 5.3 Sensitivity to changes in sample size

**Figure 5.**
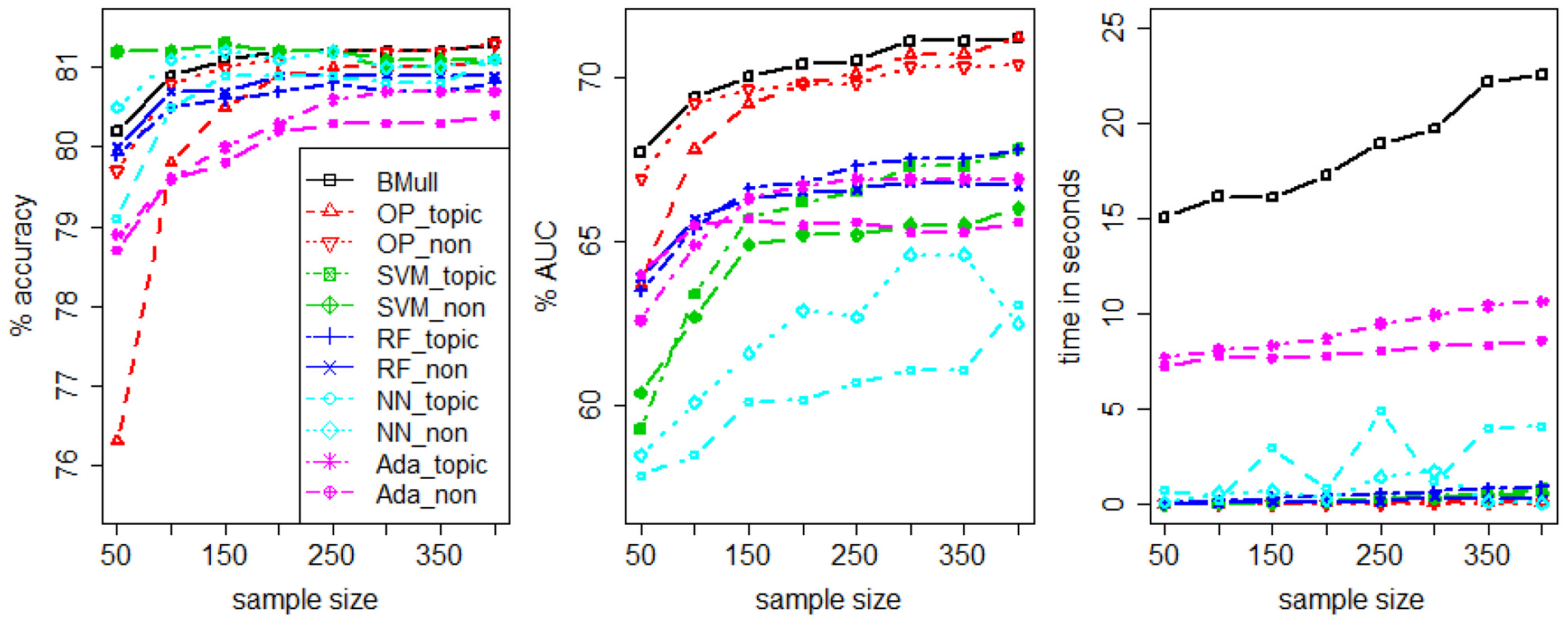
Prediction accuracy, AUC, and time complexity over different sample sizes using resampled data for depression-related drug 4.

In the previous subsections, we fix the sample size of the training set at 50. Here, we evaluate the performance of the methods by varying the sample size, and visualize how the performance and time complexity change as the sample size increases in Fig. 5 using resampled data for depression-related drug 4.

We observe that, in general, the accuracy and AUC increase as the sample size increases, except for the accuracy of SVM, which stays virtually constant for different sample sizes. In terms of accuracy, the proposed BMull is less sensitive to the sample size change as opposed to OP_topic, NN_topic and Ada, and remains very competitive for both small and large sample sizes. In terms of AUC, BMull is clearly the best while NN and SVM_non are the worst. A further examination reveals that SVM_non predicts almost all observations to be 1 in this analysis when using 0.5 as the cutoff, and so it is not able to differentiate between good and bad ratings. This also explains why its accuracy is nearly constant and its AUC is one of the lowest among all the methods.

In the third panel of Fig. 5, we report the computation times of various methods across different sample sizes. Given that our Bayesian method involves MCMC, we anticipate its computation time to be longer than that of other methods. Specifically, for the sample sizes we tested, OP, SVM, and RF all took <1 s to run, while our method required longer. Nonetheless, our method remains computationally affordable, with the longest running time clocking in at <25 s.

### 5.4 Model interpretation and insights

In addition to improved predictive power, our method is able to produce interpretable results to aid our understanding. Here, we use depression-related drug 2 on the satisfaction measure as an example and report model estimation results including posterior mean, and 95% credible region in Table 9. Unlike frequentist LASSO (Tibshirani 1996), Bayesian variable selection methods do not set any coefficient to be exactly 0. Thus, to identify important topics, we calculate the ratio between a Bayesian estimate and the corresponding frequentist estimate using an ordered probit model for each regression parameter. We use 0.5 as a cutoff for the ratio (Carvalho *et al.* 2010; Castillo *et al.* 2015; Li and Pati 2017), i.e. topics with ratio >0.5 are regarded as informative topics. Our Bayesian model identifies two informative topics for the satisfaction rating: topics 1 and 2 contribute negatively to the satisfaction rating, while other topics and prior_sentiment are regarded as irrelevant. To further understand those topics and how they contribute to the rating, the top 20 words for each topic along with a brief summary and some representative reviews for each topic are provided in Supplementary Material. To list a few of the findings: topic 1 has the largest (negative) coefficient, and it mainly discusses about the side effects of the drug. In contrast, the coefficient for topic 3 is very close to 0. A closer examination shows that this topic covers some side effects (ringing in the ear) and some mild treatment effects (helped concentrate). In practice, if patients could provide their preference about each treatment effect and tolerance level toward different kinds of side effects, our interpretable model would lead to a better understanding of the drug recommendation process and facilitate the communication between patients and doctors.

**Table 9.** BMull model parameter estimates for depression-related drug 2 on the satisfaction measure.

Variable	Bayesian estimate	Probit estimate	Ratio	95% Credible region
Female	−0.123	−0.120	1.025	(−0.446, 0.08)
Age	−0.553	−0.272	2.033	(−0.730, −0.370)
${\text{Age}}^{2}$	0.030	0.012	2.5	(0.010, 0.050)
Treatment	0.125	0.395	0.316	(−0.060, 0.378)
${\text{Treatment}}^{2}$	0.002	−0.045	0.044	(−0.044, 0.039)
Topic 0	−0.064	−0.325	0.197	(−0.397, 0.203)
Topic 1	−0.345	−0.602	0.573	(−0.753, −0.014)
Topic 2	−0.054	0.005	10.8	(−0.313, 0.145)
Topic 3	−0.003	0.024	0.125	(−0.244, 0.232)
Topic 4	0.090	0.360	0.25	(−0.134, 0.408)
Topic 5	0.075	0.312	0.240	(−0.135, 0.372)
Topic 6	0.133	0.608	0.219	(−0.093, 0.477)
Prior_sentiment	0.005	−0.705	0.007	(−0.325, 0.334)

## 6 Discussion

We proposed a BMul approach for drug recommendation. A salient feature of the proposed BMul is that multiple measures are modeled simultaneously. Thus, the models can borrow information from each other and utilize information more efficiently. For illustrative purposes, we use patient information and topic information extracted from patient review contents for model construction. we consider BMul with/without constant intercepts across tasks. To filter out nonrelevant information, especially when the number of potential predictors is large, we further propose BMull (i.e. BMul with LASSO). Such added variable selection functionality will be especially useful in situations when practitioners have an access to extra patient information such as those obtained from electronic medical records or their gene-level information.

In general, BMull performs better than a list of benchmark methods in terms of accuracy and AUC. We note that for some data examples considered, the improvement may not be as big as one wishes. As the field of supervised learning has matured, the improvement in performance has become smaller, as the state-of-the-art models have already achieved high accuracy levels on many benchmark datasets. While there is still room for improvement, it has become increasingly challenging to make significant gains in prediction performance, and more focus is shifting toward improving the interpretability, efficiency, and robustness of the model, which are indeed the main advantages of our method. Recall that our method can offer interpretable results, work efficiently for small-sized data, and is robust to possibly noninformative covariates, besides its competitive performance.

Due to the nature of the data collected, besides patients’ reviews, only patients’ basic information such as age and gender is available, while their actual symptoms are unknown. If more detailed clinical data, such as patients’ medical history and symptoms, are available, better recommendations could be made. In such situations, our method can easily incorporate them as extra features. Further, the variable selection feature of our model can tease out the irrelevant features and help make interpretable decisions. Finally, to utilize the proposed method in practice, a doctor can resort to results from our model for factors potentially affecting a patient’ drug preference. That information, in combination with our predictions and the doctor’s other assessments based on his/her expertise, could lead to a more informed decision.

## Supplementary Material

btad491_Supplementary_Data

## References

[btad491-B1] Albert JH , Chib S. Bayesian analysis of binary and polychotomous response data. J Am Stat Assoc 1993;88:669–79.

[btad491-B2] Balvert M, Patoulidis G, Patti A et al A drug recommendation system (dr. s) for cancer cell lines. arXiv, arXiv:1912.11548, 2019, preprint: not peer reviewed.

[btad491-B3] Bartlett JG , Dowell SF, Mandell LA et al Practice guidelines for the management of community-acquired pneumonia in adults. Clin Infect Dis 2000;31:347–82. 10987697 10.1086/313954 PMC7109923

[btad491-B4] Blei DM, Ng AY, Jordan MI. Latent Dirichlet allocation. J Mach Learn Res 2003;3:993–1022.

[btad491-B5] Bobadilla J , Ortega F, Hernando A et al Recommender systems survey. Knowl Based Syst 2013;46:109–32.

[btad491-B6] Carvalho CM , Polson NG, Scott JG et al The horseshoe estimator for sparse signals. Biometrika 2010;97:465–80.

[btad491-B7] Castillo I , Schmidt-Hieber J, van der Vaart A. Bayesian linear regression with sparse priors. Ann Stat 2015;43:1986–2018.

[btad491-B8] Geman S , Geman D. Stochastic relaxation, Gibbs distributions, and the Bayesian restoration of images. IEEE Trans Pattern Anal Mach Intell 1984;6:721–41. 22499653 10.1109/tpami.1984.4767596

[btad491-B9] Lakiotaki K, Tsafarakis S, Matsatsinis NF. Uta-rec: a recommender system based on multiple criteria analysis. In: *Proceedings of the 2008 ACM Conference on Recommender Systems*, Lousanne, Switzerland, 23rd-25th October 2008, 219–26. Association for Computing Machinery, New York, NY, United States

[btad491-B10] Lakiotaki K , Matsatsinis NF, Tsoukias A et al Multicriteria user modeling in recommender systems. IEEE Intell Syst 2011;26:64–76.

[btad491-B11] Li H , Pati D. Variable selection using shrinkage priors. Comput Stat Data Anal 2017;107:107–19.

[btad491-B12] Liao X , Carin L. Radial basis function network for multi-task learning. In: *NIPS’05: Proceedings of the 18th International Conference on Neural Information Processing Systems*, 2005, 792–802. Vancouver British Columbia Canada, December 5 - 8, 2005. US, MIT Press.

[btad491-B13] Lin Y-K , Chen H, Brown RA et al; Florida State University. Healthcare predictive analytics for risk profiling in chronic care: a Bayesian multitask learning approach. MIS Q 2017;41:473–95. 10.25300/MISQ/2017/41.2.07

[btad491-B14] Obozinski G, Taskar B, Jordan M. Multi-task feature selection. Technical report. University of California, Berkeley, 2006.

[btad491-B15] Obozinski G , Taskar B, Jordan MI et al Joint covariate selection and joint subspace selection for multiple classification problems. Stat Comput 2010;20:231–52.

[btad491-B16] Palanivinayagam A , Sasikumar D. Drug recommendation with minimal side effects based on direct and temporal symptoms. Neural Comput Appl 2020;32:10971–8.

[btad491-B17] Park T , Casella G. The Bayesian lasso. J Am Stat Assoc 2008;103:681–6.

[btad491-B18] Rao TVN, Unnisa A, Sreni K. Medicine recommendation system based on patient reviews. Int J Sci Technol Res 2020;9:3308–12.

[btad491-B19] Romagnoli KM , Nelson SD, Hines L et al Information needs for making clinical recommendations about potential drugdrug interactions: a synthesis of literature review and interviews. BMC Med Inform Decis Mak 2017;17:21. 28228132 10.1186/s12911-017-0419-3 PMC5322613

[btad491-B20] Silver DL , Poirier R, Currie D et al Inductive transfer with contextsensitive neural networks. Mach Learn 2008;73:313–36.

[btad491-B21] Stark B , Knahl C, Aydin M et al A literature review on medicine recommender systems. Int J Adv Comput Sci Appl 2019;10:6–13.

[btad491-B22] Sun L, Liu C, Guo C et al Data-driven automatic treatment regimen development and recommendation. In: *KDD ’16 Proceedings of the 22nd ACM SIGKDD International Conference on Knowledge Discovery and Data Mining*, San Francisco, California, United States. August 13-17, 2016, 1865–74. Association for Computing Machinery, New York, NY, United States.

[btad491-B23] Tibshirani R. Regression shrinkage and selection via the lasso. J R Stat Soc Series B 1996;58:267–88.

[btad491-B24] Xue Y , Liao X, Carin L et al. Multi-task learning for classification with Dirichlet process priors. J Mach Learn Res 2007;8:35–63. PMC3754453 23990757

[btad491-B25] Zhang Q, Zhang G, Lu J et al A framework of hybrid recommender system for personalized clinical prescription. In: 10th *International Conference on Intelligent Systems and Knowledge Engineering*, Taipei, Taiwan, November 24-27, 2015. Institute of Electrical and Electronics Engineers (IEEE), Newyork, NY, United States.

[btad491-B26] Zhang Y , Yang Q. A survey on multi-task learning. IEEE Trans Knowl Data Eng 2022;34:5586–609. 10.1109/tkde.2020.3045924 PMC10619966 37915376

[btad491-B27] Zhang Y , Zhuang Y, Wu J et al Applying probabilistic latent semantic analysis to multi-criteria recommender system. AI Commun 2009;22:97–107.

