# Supplementary material for: Bayesian multitask learning for medicine recommendation based on online patient reviews
Source: Bioinformatics. 2023 Aug 8;39(8):btad491. doi: 10.1093/bioinformatics/btad491 (PMC10425196; doi:10.1093/bioinformatics/btad491)
Supplement: btad491_Supplementary_Data [file btad491_supplementary_data.pdf]

Supplementary Material for Bayesian Multi-task Learning for Medicine Recommendation Based on Online Patient Reviews

S1 Technical background on Topic Modeling

Here, we explain the basic idea and provide some technical background of a widely used topic modeling method: the latent Dirichlet allocation (LDA) developed by Blei et al. (2003). LDA is a generative probabilistic model that aims to find clusters of words that tend to appear together. The basic idea is that documents (reviews) can be represented by mixtures of latent topics, where each topic is characterized by a word distribution and each review is treated as one document. The LDA model produces two matrices: the word distribution for each topic and the coverage of the topics in each document. Intuitively, a topic implies a distribution on words, so words associated with that topic have a high likelihood of occurring. Such relationship between topics and word distributions is described by the first matrix. Second, a document can discuss several topics; the second matrix tells us how much a given document is related with a particular topic.

Before applying the LDA model, the documents usually go through several pre-processing steps, including stemming, lemmatization and stop words removal. Stemming and lemmatization transforms words into their base form and stop words refers to words that carry little information, such as "I", "and", "thus", etc. These pre-processing steps help to consolidate words that share similar meaning and reduce the total number of unique words.

We now define the components needed to conduct an LDA analysis:

- A word is the basic unit of a document, defined to be an item from a vocabulary indexed by $1, 2, 3, \ldots V$. Words are represented by unit basis vectors such that only

one component is equal to one and all other components equal to zero.

- A document (in our case a review) is a sequence of words denoted by $d_i = (w_{i1}, \ldots, w_{iN_i})$, where N_i is the total number of words in document i and w_{in} is a unit basis vector for the nth word in document i.

- A corpus is a collection of M documents denoted by $D = \{d_1, \ldots, d_M\}$.

Suppose there exists a total of K topics, then LDA assumes the following generative process for the ith document:

- The number of words N_i follows a Poisson distribution $Poi(\xi)$

- The topic distribution $\pi_i = (\pi_{i1}, \ldots, \pi_{iK})^T$, with $\sum_k \pi_{ik} = 1$, follows a Dirichlet distribution $Dir(\eta)$ where η is a vector parameter of length k to be estimated.

- To generate the nth word for document i, we first choose a topic: $z_{in} \sim Multinomial(\pi_i)$, then choose a word $w_{in} \sim Multinomial(\theta_{z_{in}})$ where $\theta_{z_{in}}$ is a column vector parameter of length V.

The probability of generating all the words in document i is simply the product of the word distribution in each topic over all the words $\prod_{n=1}^{N_i} p(z_{in}|\pi_i)p(w_{in}|\theta_{z_{in}})$ multiplied by the topic coverage $p(\pi_i|\eta)$, the probability density function (pdf) for a Dirichlet distribution with parameter η. Given the parameters η and $\theta = (\theta_1, \ldots, \theta_K)$, the joint distribution of a topic mixture π, a set of N_i topics $z_i = (z_{i1}, \ldots, z_{iN_i})$, and a set of N_i words d_i is:

$$p(z_i, d_i, \pi_i|\eta, \theta) = p(\pi_i|\eta)\prod_{n=1}^{N_i} p(z_{in}|\pi_i)p(w_{in}|\theta_{z_{in}})$$

Integrating over π_i and summing over z_i, we obtain the marginal distribution of a document:

$$p(d_i|\eta, \theta) = \int p(\pi_i|\eta)\sum_{z_{in}}\prod_{n=1}^{N_i} p(z_{in}|\pi_i)p(w_{in}|\theta_{z_{in}})d\pi_i$$

Finally, taking the product of the marginal probabilities of each document, we obtain the probability of generating a corpus D based on the parameter η and θ:

$$p(D|\eta,\theta) = \prod_{i=1}^{M} \int p(\pi_i|\eta) \sum_{z_{in}} \prod_{n=1}^{N_i} p(z_{in}|\pi_i) p(w_{in}|\theta_{z_{in}}) d\pi_i$$

The parameters η and θ can be found by maximizing the above likelihood.

S2 Tables for topic modeling results

In this section, we show the topic modeling results on depression related drug 2. In Table S1, we list the top 20 words for each topic along with a brief summary. In Table S2, we list some representative reviews for each topic to provide further insights.

Topic	Top 20 words	Summary
0	'suffer', 'suicid', 'quot', 'wors', 'pound', 'antidepress', 'gain', 'sever', 'switch', 'happi', 'celexa, 'anger', 'couldn', 'major', 'chronic', 'zoloft', 'prozac', 'make', 'dont', 'recommend'	Make my depression worse
1	'brand', 'generic', 'away', 'sever', 'attack', 'tell', 'need', 'quot', 'loss', 'hope', 'wake', 'review', 'coupl', 'panic', 'scar', 'recommend', 'love', 'happi', 'get', 'hour'	loss of energy, the brand name version would be better than the generic
2	'generic', 'get', 'drink', 'alcohol', 'switch', 'actual', 'wasn', 'point', 'normal', 'wonder', 'issu', 'prozac', 'need', 'tire', 'symptom', 'complet', 'problem', 'med', 'peopl', 'wake'	get vertigo when I drink alcohol
3	'motiv', 'pain', 'love', 'person', 'ear', 'ring', 'daili', 'abl', 'suffer', 'haven', 'major', 'wonder', 'cri', 'come', 'drive', 'amaz', 'caus', 'loss', 'concentr', 'med'	Ringing in the ear; helped concentrate
4	'zoloft', 'extrem', 'lexapro', 'thought', 'morn', 'prozac', 'emot', 'appetit', 'sexual', 'gain', 'suicid', 'improv', 'drive', 'mouth', 'switch', 'long', 'antidepress', 'get','brain','fall'	Have more energy; loss of appetite
5	'smoke', 'quit', 'pill', 'headach', 'morn', 'concentr', 'focus', 'loss', 'troubl', 'nausea', 'appetit', 'decreas', 'hour', 'crave', 'suffer', 'caus', 'improv', 'fine', 'love','abl'	Help quit smoking
6	'quot', 'headach', 'problem', 'happi', 'pill', 'normal', 'head', 'say', 'horribl', 'nausea', 'away', 'result', 'long', 'twice', 'exercis', 'begin', 'insomnia', 'occasion', 'high', 'final'	Happy with the medicine; started exercise, occasional nausea, insomnia and headache.

Table S1: Top words and topic summaries for depression related drug 2.

Topic	Representative (partial) review
0	Its not helping much seems like its making my depression worse. I just don't know what to take anymore
1	I have beeen on the generic form of Wellbutrin for 2 days now BIG mistake to switch from Pristiq to this. This medication has made me feel like a zombie I feel achy hungover tired depressed and have lost all energy and will to exercise. I have read many reviews stating that the generic and brand name versions are very very different. I would be willing to bet that I would not have had this negative experience if my doctor had prescribed the the brand name version instead of the generic
2	Dry mouth and a little dizziness the morning after drinking alcohol
3	I have ringing in my ears; I started taking this 3 weeks ago for depression anxiety. It really helped my concentration but it also kept me up all night every night
4	I have been on paxil prozac and zoloft and they never worked for me I have been on the Bupropion for 8 days and so far I like it. I had no energy for my 3 year old or my fiance and work was draining me every day. I have more energy which is what I was going for and I don't feel tired. I do take a 25mg of Trazadone to help me sleep and it seems to work good with the Bupropion. I have noticed a loss of appetite which isn't bad but when I'm busy I forget to eat.
5	I have been taking Bupropion HCI XL 300 mg for about 10 years. It is the only medication that works for me with the least amount of side effects dry mouth and itching I do take an allergy pill as well. I don't walk around crying for no reason or get upset over little things I don t have thoughts of wanting to die anymore I quit smoking cold turkey 3 years ago and have remained a non smoker. This drug has been great for me
6	It took a few weeks but I am so glad I got help. I started to feel better had more energy where before I could barely make it through the day. I lost weight and started feeling more positive. I experienced an increase in libido and a slightly decreased appetite I started exercising and although I've suffered some additional setbacks. For now I am very happy with this medicine.

Table S2: Representative reviews for each topic for depression related drug 2.

References

Blei, D. M., Ng, A. Y., and Jordan, M. I. (2003). Latent dirichlet allocation. *Journal of Machine Learning Research*, 3:993–1022.
